# Co‐Designing a Model of Brilliant Care for Older People

**DOI:** 10.1111/jocn.70049

**Published:** 2025-08-01

**Authors:** Ann Dadich, Rachael Kearns, Ben Harris‐Roxas, Katherine Boydell, Peter Gonski, Friedbert Kohler, David Lim, Éidín Ní Shé, Carmen Amato, Imelda Gilmore, Brian Lane, Jane Mears, Frank Schaper, Rosslyn Sleeman, Elise Tcharkhedian, Varsha Tembe, Shannon Azzopardi, Kim Delbaere, Tamar Krebs, Elizabeth Paterson‐Finlay, Danielle Ni Chroinin

**Affiliations:** ^1^ School of Business Western Sydney University Penrith New South Wales Australia; ^2^ Institute for Culture and Society Western Sydney University Penrith New South Wales Australia; ^3^ School of Population Health University of New South Wales Sydney New South Wales Australia; ^4^ Black Dog Institute Randwick New South Wales Australia; ^5^ South Eastern Sydney Local Health District & University of New South Wales, Sydney Taren Point New South Wales Australia; ^6^ HammondCare Health, South Western Sydney Local Health District & University of New South Wales, Sydney Prairiewood New South Wales Australia; ^7^ University of Technology Sydney Ultimo New South Wales Australia; ^8^ Royal College of Surgeons in Ireland Dublin Ireland; ^9^ South Western Sydney Local Health District Liverpool New South Wales Australia; ^10^ Western Sydney University Penrith New South Wales Australia; ^11^ Consumer and carer representatives, New South Wales Australia; ^12^ Blacktown Council Aged Care Committee Blacktown New South Wales Australia; ^13^ Garrawarra Care Centre Garrawarra Centre Waterfall New South Wales Australia; ^14^ Neuroscience Research Australia University of New South Wales, Sydney Randwick New South Wales Australia; ^15^ Group Homes Australia North Sydney New South Wales Australia; ^16^ Banksia Villages Ltd Broulee New South Wales Australia; ^17^ Liverpool Hospital South Western Sydney Local Health District & University of New South Wales, Sydney Liverpool New South Wales Australia

## Abstract

**Aim:**

This study aimed to co‐design a model of brilliant care for older people that provides clear, actionable principles to guide how brilliant care for older people can be realised.

**Background:**

As the demand for and international importance of care for older people grows, so too does the negative discourse about care for older people. This ongoing focus on deficiencies can have implications for patients, carers, clinicians, health services, and policymakers, overshadowing opportunities for innovation and positive change.

**Design:**

Experience‐based co‐design informed this study, grounded in the lived experiences of key stakeholders.

**Methods:**

Three scaffolded co‐design workshops were facilitated, involving lived experience experts, managers, professionals, clinicians, and an academic (*n*= 13). The data collected during these workshops were analysed using a qualitative descriptive method and documented according to COREQ guidelines to optimise rigour and transparency.

**Results:**

The participants co‐designed a model of brilliant care for older people, comprising principles to promote connection and innovation. To promote connection, the model includes protecting staff member time to deliver meaningful care and demonstrating that everyone matters. To promote innovation, it encourages role flexibility, curiosity, small improvements, and the recognition of brilliant practices.

**Conclusions:**

This article presents a co‐designed model of brilliant care for older people, incorporating principles of connection and innovation that can be enacted through simple, resource‐efficient practices.

**Relevance to Clinical Practice:**

For those who manage and deliver care for older people, the model encompasses simple, accessible, and cost‐effective principles to: positively deviate from norms within the sector, offering care to older people; and to deliver brilliant care for older people. Furthermore, given that the model was co‐designed with lived experience experts, managers, professionals, and clinicians, its principles are imbued with their experiential insights, which served to bring particular priorities to the fore.

**Patient or Public Contribution:**

The co‐designers, who included lived experience experts, were invited to participate in workshops to co‐design a model of brilliant care for older people, during which they discussed and critiqued the findings constructed from the data and co‐designed the model.

**Summary**

- What does this paper contribute to the wider global clinical community?
  ○ To promote brilliant care for older people, this article presents a co‐designed model of brilliant care for older people, comprised of principles to promote connection and innovation.
  ○ To promote connection, the model includes protecting staff member time to deliver meaningful care and demonstrating that everyone matters; and to promote innovation, it encourages role flexibility, curiosity, small improvements and the recognition of brilliant practices.
  ○ For those who manage and deliver care for older people, the model encompasses simple, accessible, and cost‐effective principles to positively deviate from norms within the sector and deliver brilliant care for older people.

## 1 Introduction

Despite the growing demand for and international importance of care for older people (United Nations 2001; World Health Organization 2022), many older people's services have a limited capacity to offer the care that older people and their family members need and want (Baines et al. 2021; Farr‐Wharton et al. 2021). While this was magnified during the COVID‐19 pandemic (Chan et al. 2021), the challenges in care for older people were evident long before. For example, a report on social care for older people in the United Kingdom found the ‘social care system… is struggling to meet the needs of older people’ (Humphries et al. 2016, p. 3). And in Australia, a royal commission exposed suboptimal care in the sector offering care to older people and espoused a need to rethink how we offer care to older people (Batchelor et al. 2020; Royal Commission into Aged Care Quality and Safety 2020).

Limited sector ability to meet the needs and preferences of older people and their family members can create personal, social, and economic challenges. On a personal level, substandard care for older people can compromise the dignity and well‐being of older people and their family members, potentially leading to neglect and abuse, decreasing quality of life (St Clair et al. 2022). Priority populations—including people of culturally and linguistically diverse backgrounds, people who identify as Indigenous, and people who experience poverty (Hill 2022)—are disproportionately affected, exacerbating inequities in care for older people. On a social level, substandard care for older people can exacerbate the burden of care on family members as well as social isolation (Boucher et al. 2019). On an economic level, the health system can suffer (University of Queensland 2020), as poor care can increase the need for emergency care and hospitalisation at a more advanced state of poor health, delay hospital discharges, increase burnout among staff members, and reduce workforce capacity (Gullick and Islam 2023; Low et al. 2022).

Despite considerable evidence on the challenges in care for older people (Strivens and Stirling 2023), recent research suggests that brilliance in care for older people happens (Dadich et al. 2023). Through interviews with individuals recognised for their brilliant care for older people, researchers found that brilliant care for older people is characterised by: a deep understanding of the older person; recognition that caring for older people is ‘more than a job’; innovative practices; and empowering staff members with the freedom to reprioritise what they did and how they did it.

The care system for older people is facing a critical challenge: it is consistently failing to meet the needs and preferences of older people and their families. This shortfall has led to widespread consequences—including compromised dignity and well‐being, increased social isolation, and greater strain on healthcare systems. To address this urgent issue, it is important to reconceive care for older people that centres on the lived experiences of older people and the people who support them.

Building on the aforesaid insights (Dadich et al. 2023), the aim of this study was to co‐design a model of brilliant care for older people to clarify how brilliant care for older people can be realised in practice. This was achieved by facilitating co‐design workshops with individuals who had a stake in the study.

This article commences with an overview of brilliant care and then clarifies how a model of care is here understood. Following a description and justification of the methodology, the model of brilliant care is then explicated. The article then concludes by summarising the key findings and expounding the associated implications for scholars and practitioners.

### 1.1 What Is Brilliant Care?

Brilliant care refers to care that exceeds expectation, bringing joy and delight to those who experience or witness it (Dadich et al. 2023; Fulop et al. 2018). It is not tied to specific health outcomes but is a relational experience. It can be unconventional and serendipitous, diverging from business‐as‐usual within a service or a sector. It is interpersonal, uplifting, inspiring, and/or energising (Dadich et al. 2018). As such, aspiring for brilliance in care goes beyond meeting or exceeding performance indicators.

This focus on brilliance is important because it helps to counterbalance the dominant narrative that highlights the problems and deficiencies in the sector. With few exceptions (King and Miller 2021; Minney and Ranzijn 2016), discourse around care for older people is often framed negatively. Research tends to focus on ‘issues’ (Seaward et al. 2022), ‘gaps’ (Blackberry and Morris 2023), ‘stigma’ (Manchha et al. 2023), ‘problems’ (Ervin et al. 2012), and ‘adverse incidents’ (St Clair et al. 2022). Similarly, the media presents accounts of ‘suffering’ (Blackwood 2022), potentially contributing to ‘a distant, often decontexualised or trivialised, and non‐representative understanding of what aged care… is like’ (Thomson et al. 2022).

While identifying and addressing shortcomings in care for older people is important, an overemphasis on problems can create additional challenges. This focus can silence patients' and carers' positive experiences with the care they receive, deter help‐seeking behaviours, and hinder subsequent access to timely care (Barney et al. 2009). It can also unfairly stereotype clinicians and health services as part of a systemic failure (Butterill et al. 1992) and diminish learning opportunities and innovation (Ashby et al. 1999). Furthermore, it can divert policymaker support and public funds towards poor clinical and managerial practices instead of supporting positive developments. Raising the profile of positive experiences is critical because positivity begets positivity (Lyubomirsky et al. 2005).

In the context of care for older people, brilliance is epitomised by the previously mentioned constituents—namely, a deep understanding of the older person; recognition that caring for older people is ‘more than a job’; innovative practices; and having permission to reprioritise what you do and how you do it. Although explicated elsewhere (Dadich et al. 2023), in brief, brilliant care for older people involves an active orientation towards the older person's needs and preferences, facilitating connections with family members to support care. It also requires an understanding that caring for older people is not a mere job, but an opportunity to form caring and meaningful relationships. Given that delivering care to older people can be difficult, it is important to invest in staff members via opportunities for personal and professional development—staff members who feel cared for are better able to provide high‐quality care. Additionally, brilliant care for older people involves the initiative to trial novel practices that have the potential to make a positive difference to older people. Such innovation is often prompted by the need to solve a problem, often with limited resources. Finally, brilliant care for older people requires the authorisation to exercise agency, allowing for flexibility rather than rigid task completion. This freedom creates space for conversations and relationships to flourish, which are mutually enjoyable and valued by older people and their family members.

While the aforesaid constituents are informative, they lack guidance on how they might be operationalised in practice. For instance, it is unclear what a deep understanding of the older person might involve. Similarly, there might be varied (if not mis‐)understandings of how staff members are afforded the permission to reprioritise. Thus, practical guidance is required to not simply rethink, but rather reimagine care for older people in ways that are feasible and sustainable (Royal Commission into Aged Care Quality and Safety 2020). This might be in the form of a model of brilliant care for older people.

### 1.2 What Is a Model of Care?

The literature on models of care suggests three key points. First, there is no universallyagreed definition. For instance, they are sometimes depicted as a schematic, specifying tasks that are performed in a linear manner (Muntinga et al. 2012). Models of care are also expressed as ‘pillars’ that collectively characterise ‘healthcare service delivery, role determination, governance and development of role relationships with patients, family and the healthcare team’ (Booker et al. 2016, p. 137). Second, implementing models of care can be hindered by myriad factors. At a macro level, these can include policies that are not conducive to the model (Threapleton et al. 2017); at a meso level are limited leadership (Shin et al. 2023) and managerial support (Dunér et al. 2011); and at a micro level are insufficient staff training and experience (McArthur et al. 2021). Additionally, efforts to implement a model of care can be hampered by limited detail on the model of care. For instance, a scoping review on models of care for older people found that, of the 276 publications identified, 68.1% ‘failed to present the model of care in a clear stepwise manner to aid understanding and facilitate use elsewhere’ (Dadich et al. 2025, p. 6). Third, despite the absence of a single definition, the literature typically portrays models of care as processual, rather than an isolated event. They represent ‘the organisation of care’ (Luckett et al. 2014, p. 2), outlining ‘best practice care and services for a person, population group or patient cohort as they progress through the stages of a condition, injury or event’ (NSW Agency for Clinical Innovation 2013, p. 3).

Building on this literature and given the focus of this study—care for older people—which spans different contexts (e.g., residential facilities, homes, short‐term services), a model of care is here understood as a set of guiding principles or a specific philosophy of care. As such, the intention of this study was not to co‐design a definitive structure to dictate services, the tasks therein, roles, responsibilities, and governance arrangements—but rather, to co‐design principles to enact the constituents of brilliant care for older people.

## 2 Methods

### 2.1 Study Design

Following approval from the relevant human research ethics committee (approval number: 2021/ETH01195), three workshops were facilitated with stakeholders to co‐design a model of brilliant care for older people, informed by previous research (Dadich et al. 2023).

This study was guided by experience‐based co‐design, given its focus on creating better quality experiences and models of care (Dimopoulos‐Bick et al. 2018; Donetto et al. 2015). Although experience‐based co‐design has principally been used for quality improvement in health services, its promise has been recognised for intervention development (Green et al. 2020). The co‐design activities described in this article encompass both purposes by identifying aspects of brilliant care for older people, as experienced by consumers and carers, and co‐designing a model of brilliant care for older people to take forward.

While there is no universal understanding of what constitutes co‐design (Masterson et al. 2022), co‐design was here defined as a process of actively working with diverse experts—including people with lived experience—to ‘design… solutions to a pre‐specified problem’ (Vargas et al. 2022, p. 1). The co‐design process reflected Bogomolova et al. (2021) approach. Specifically, workshops were facilitated to generate creative solutions by reviewing various ideas and principles, deciding on the most attainable, and using these to co‐design the model of brilliant care for older people. The overall goal of the co‐design workshops was to enhance the design and delivery of care for older people.

### 2.2 Participants

To diversify the expertise represented in the study, an email invitation to participate in the workshops was circulated via the Age and Ageing Clinical Academic Group of Maridulu Budyari Gumal—also known as the Sydney Partnership for Health, Education, Research and Enterprise (2024). SPHERE is an Australian, nationally funded partnership comprised of 16 partners, including local health districts, speciality health networks, universities, and medical research institutes. Within SPHERE are 16 clinical academic groups, each focused on a particular interest, including (but not limited to) Aboriginal health and well‐being, the early determinants of life, palliative care and—of particular relevance to this study—age and ageing. Given the Age and Ageing Clinical Academic Group includes clinicians, service managers, academics, consumers, and carers with a shared interest in age and ageing, it was an appropriate and expansive network through which to extend the invitation. Prospective participants responded to the email invitation, after which a researcher ensured they fulfilled the eligibility criteria. Specifically, participants were required to be 18 years of age or over and conversant in English. To optimise diversity, the researchers also purposefully recruited lived experience experts who were under‐represented—namely, people who received care as an older person and/or cared for an older person.

Thirteen participants were collectively involved in the workshops. Most were female (*n* = 11) and the largest proportion received care as an older person and/or cared for an older person (*n* = 5, see Table 1)—given these intersectional identities, it was not appropriate to discretely identify these participants as a consumer or a carer, as they typically assumed both roles. Due to the limited availability of research funds, the participants with lived experience expertise did not receive recompense. Given that the greatest proportion of participants had lived experience expertise, this constitutes a strength of this study—this might be partly due to the online nature of the workshops, which offered convenience. Although not all participants were involved in all three workshops—with participation ranging from 10 to 12 participants (see Table 2)—all participants received discussion summaries and were invited to confer with the facilitators between the workshops to ensure they were not disadvantaged by their absence.

**TABLE 1:** Participants (*n* = 13).

Participant expertise	Number
Lived experience experts	5
Managers	3
Professionals	2
Clinicians	2
Academic	1
Total	13

**TABLE 2:** Workshop participation (*n* = 13).

Participant expertise	Workshop 1	Workshop 2	Workshop 3
Academic	1	1	1
Clinician	2	1	2
Lived experience expert	4	4	5
Manager	3	2	1
Professional	2	2	1
Total	12	10	10

### 2.3 Workshops

Following participants' informed consent, two researchers facilitated three 90‐minute workshops via web‐conference (for participant convenience), a month apart in August, September, and October 2022. This timing provided the facilitators and participants with an opportunity to reflect on each workshop, consider what they had learnt or hoped to learn, and prepare for the subsequent workshop. Throughout the workshops, participants were encouraged to ask questions (of the facilitators and each other) and contribute their ideas, experience, and knowledge. One facilitator actively facilitated discussion, while the other captured and presented the ideas discussed during the workshops using Miro (2023)—an online, interactive platform. Participants were invited to contact the facilitators between the workshops if there were ideas or thoughts they wanted to discuss further.

**FIGURE 1:**
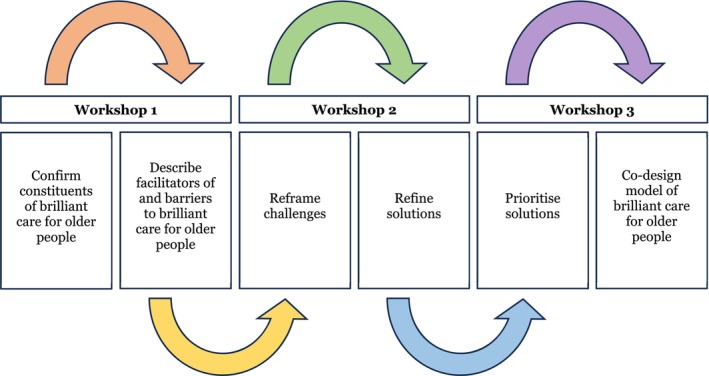
Precis of co‐design process. [Colour figure can be viewed at wileyonlinelibrary.com]

The workshops were designed to reflect an iterative process, with the second and third workshops building on the previous workshop(s) (see Figure 1). The first workshop commenced with a discussion of the previously identified constituents of brilliant care for older people (Dadich et al. 2023). Guided by a workshop schedule (see Table 3), participants were then invited to consider: the choice of language about the constituents and how clarity might be improved; which of the constituents resonated with, or surprised them and why; which of the constituents mattered most and why; how the constituents might be operationalised in practice; the factors that might help or hinder these practices; and how to promote or manage these factors. Building on this discussion, the second workshop commenced with a summary of the first workshop discussion and an invitation to participants to correct misunderstandings and/or add to the summary. For transparency, the facilitators corrected misunderstandings and added comments to the growing online dataset (using Miro) as they were offered—these included factors that hindered brilliant care for older people, like the ubiquity and lost meaning of person‐centred care, as well as managers who discouraged staff members from developing friendships with older people and their family members. Following this, and guided by a workshop schedule, participants were then invited to: consider which of their suggested principles to enact brilliant care for older people would offer the most impact for the least effort; as well as which principles should be prioritised and why. Building on this discussion, the third workshop commenced with a summary of the second workshop discussion and invited participants to correct misunderstandings and/or add to the summary. Following this, and guided by the workshop schedule, participants were invited to identify: three principles the aged sector should focus on and why; and three changes that would help to readily implement the principles and why. Although it was sometimes difficult for participants to constrain their selections, this approach served to prioritise options for the sector offering care to older people, which is typically under‐resourced.

**TABLE 3:** Workshop guide.

**Workshop 1**
What resonates with you? What surprises you? What matters most? What should a service for older people do, to demonstrate these constituents? Reflecting on your experiences, would this work? What would enable this to happen? What might hinder this, and how could these be managed? How would we know if a service for older people had these constituents – what are the signs? Which principles should we prioritise, and why? How might these be tested – where and by who? How would we know if the principles worked?
**Workshop 2**
Thinking about the potential effort and impact of each principle, which principles should we prioritise, and why? What are your top two principles and why?
**Workshop 3**
If you had to select three principles that the aged sector should focus on, what would they be and why? What are the first three changes that you would do to operationalise or implement the principle and why?

Given that participants represented different and intersecting experiences and identities, conflicting views and priorities were expected. When these manifested, the facilitators: reminded participants to demonstrate mutual respect; ‘Offer[ed] generous listening’ (McKercher 2020, p. 48), ensuring that participants had ample opportunity to express their views and have these views affirmed; and revisited participants' shared and complementary interests to collectively remain focused on a common vision—namely, to promote brilliant care for older people. The facilitators debriefed after each workshop to reflect on the discussion, how it was facilitated, and ways to improve their facilitation skills; they also synthesised the data collected for presentation at the subsequent workshop.

### 2.4 Data Analysis

Participants' contributions were summarised and synthesised from the facilitators' written notes and those documented via an online shared whiteboard platform (Miro), which participants could access and edit. Following the three workshops, recordings were purposively transcribed for further analysis (Halcomb and Davidson 2006). The authors analysed the data using a qualitative descriptive method (Colorafi and Evans 2016). This process served to clarify participant principles to operationalise the constituents of brilliant care for older people and articulate a model of brilliant care for older people. To optimise rigour and transparency, the consolidated criteria for reporting qualitative studies (COREQ, Tong et al. 2007) were followed (see File S1).

## 3 Results

### 3.1 Reflections on the Constituents of Brilliant Care for Older People

#### 3.1.1 Deep Understanding of the Older Person

The participants indicated that the constituents of brilliant care for older people largely resonated with their own experiences. In particular, they emphasised the importance of developing a deep understanding of the older person and getting to know older people through thorough (not merely clinical) assessments. Participants underscored the value of taking the time to consider what is important to them, including a person's life story, who they are, and how they have spent their life. For older people, particularly people living with dementia, knowing these personal details was essential for meaningful care. Each person's story is unique and brilliant care for older people was described as recognising and acting on those ‘little things’ that are different and important for each person:

aged care is moving into the direction that each person is different, the requirements are different, and if you address those little things for each patient it might be better for them (*lived experience expert*).

#### 3.1.2 More Than a Job

Elements of the discussion affirmed that, for care for older people to be brilliant, caring for older people needed to be regarded as more than a job. The ways that care was delivered were deemed significant and should reach beyond merely working through a set of caring tasks. To exemplify this, one participant described how assessments can be completed with older people with genuine engagement, rather than as a form of task completion. Similarly, a participant noted that nursing had become ‘very clinical’ (*lived experience expert*), with limited attention on physical touch—such, as giving patients ‘a hug’—or relationships between nurses and patients. Another participant juxtaposed this observation with a description of a facility that permeated a person‐centred ‘attitude’ (*lived experience expert*), demonstrated by mutual respect between and among staff members and patients.

#### 3.1.3 Innovative Practices

Participants considered innovative practice as essential for brilliant care for older people. Given their familiarity with the older people they supported and their expertise, staff members were said to be ‘full of solutions’ (*lived experience expert*) that can improve older people's quality of life. Participants emphasised the need for staff members to be empowered to exercise initiative, rather than be complacent by accepting current practices without considering opportunities for improvement:

That's how brilliant models often start… someone says, ‘Can we do this?’ (*clinician*).

While participants recognised the constrains of limited funds, they noted that innovation can be prompted by limited access to resources. They were mindful that necessity can foster innovation, whereby people are required to exercise resourcefulness. For instance, one participant described how different ideas were shared across interagency forums and subsequently adapted in different services for feasibility:

As a society, when we have problem… we immediately look to money… to solve the problem for us… innovation… relies on the way… people do things (*lived experience expert*).

#### 3.1.4 Permission to Reprioritise

Participants recognised the significance of encouraging staff members to reprioritise what they did and how they did it. Given limited resources, some suggested it was important to ensure that care for older people was everyone's business, whereby everyone had a role in delivering and optimising care. For instance, all staff members, including cooks, cleaners, and staff members who held non‐clinical roles, could be trained to provide care in ways that improved the care environment. Similarly, participants highlighted the importance of integrating physical activity into daily routines, making it a shared responsibility across all staff roles. By fostering a culture where everyone encourages movement, services can better support mobility through simple actions, such as walking to meals. By shifting the mindset that care for older people was solely the purview of clinicians and personal care staff members, limited resources could be used more effectively and efficiently:

There's never enough time, there's never enough resources, there's never enough staffing and if we continue down that route of never enough, we're never going to change anything… I think… reframing the way we look at our facilities, our environments, our homecare… when you look at aged care… the AINs [assistants in nursing], the registered nurses, the cooks, the cleaners; there's so much staff. The problem is that we've created silos and not an industry where everyone is trained to give care… people say that we don't have enough time, we don't have enough staff. This is what we have. And so it's how do we shift our mindset in using what we have? (*professional*).

### 3.2 Challenges and Principles

Having indicated that the constituents of brilliant care for older people resonated with them, the participants were invited to consider the challenges that stymied such care, as well as the principles to address these to ultimately inform the model of brilliant care for older people. To optimise feasibility, participants were encouraged to focus on principles that were likely to be feasible.

Guided by participant advice, the facilitators classified the challenges and principles into higher‐order categories and invited participants to review their appropriateness (see Table 4). Although interrelated, the challenges pertained to: risk aversion; limited personal agency; impersonal care; and low morale and energy. To address these, the participants identified principles that support brilliant care for older people, which the authors subsequently sorted into those that promote connection and those that promote innovation. The former aligned with the constituents of a deep understanding of the older person and recognition that caring for older people is ‘more than a job’; while the latter reflected the constituents of innovative practices and having permission to reprioritise what you do and how you do it. In the following sections, the challenges and associated principles are elucidated.

**TABLE 4:** Challenges to and principles for brilliant care for older people.

Constituents	Challenges	Principles
Deep understanding of the older person	Risk aversion This is too much concern risk aversion, reflected by a risk‐averse culture Staff members are discouraged from forming friendships with older people and their family members Depersonalised care There is a limited understanding of the older person There is too much focus on completing the same tasks for all older people, without tailoring care to individual preferences There is a clear focus on clinical needs with limited attention to interpersonal relationships, including families There is a misperception that there is limited time to connect with older people Time is needed to better understand older people, beyond standard questionnaires Limiting personal agency Things are done for rather than with older people There is a focus on completing tasks rather than enabling older people Physical environments do not always support older people	Promoting connection Develop and sustain dialogue with older people and within their circle of care Use practical methods to ensure older people are at the centre of decision‐making with family members and staff members Offer the dignity of choice to older people Appreciate individuals – show them that they matter Introduce meaningful assessments and processes that enable staff members to better understand older people earlier Be focused on older people and their family members Demonstrate that everyone matters in care for older people: older people, family members and staff members, regardless of position Encourage human touch Protect time Use scholarships, awards, funds, days of leave, and other forms of recognition to acknowledge brilliant care and better outcomes among: industry and government; as well as individuals and teams Reduce the precarity of secure employment Be empathic by: ○ Recognising the experience of grief and loss ○ Creating affective encounters during transitions ○ Remembering that first impressions count
Caring for older people is ‘more than a job’	Low morale and energy Staff members are often worn down	
Innovative practices	Risk aversion This is too much concern risk aversion, reflected by a risk‐averse culture Staff members are not permitted to trial different practices Staff members are discouraged from forming friendships with older people and their family members	Promoting innovation Ask questions beyond the standard (clinical) assessments Encourage role flexibility and skill development Share different ways of engaging with older people, service offerings and models of care and normalise this sharing practice—for instance, offer opportunities for staff members to exchange information, reflect, explore caring issues, discuss possible solutions and identify the resources required Do not take no for an answer Engage with (rather than avoid) risk Ensure the physical environment suits the needs and preferences of older people and family members Foster a culture of innovation by: ○ Encouraging and rewarding initiative ○ Promoting curiosity and questioning why ○ Thinking differently ○ Letting go of established practices ○ Hiring for culture ○ Learning from other sectors (e.g., leisure) ○ Normalising initiative ○ Introducing small improvements, particularly those that are not resource‐intensive ○ Introducing intergenerational activities Encourage physical activity in its varied, incidental and bite‐sized forms Ensure performance assessment systems serve to recognise brilliant practices and identify opportunities for quality improvement
Permission to reprioritise	Risk aversion This is too much concern risk aversion, reflected by a risk‐averse culture Staff members are not permitted to trial different practices Staff members are discouraged from forming friendships with older people and their family members Depersonalised care There is too much focus on completing the same tasks for all older people, without tailoring care to individual preferences There is a clear focus on clinical needs with limited attention to interpersonal relationships, including families There is a misperception that there is limited time to connect with older people Time is needed to better understand older people, beyond standard questionnaires Limiting personal agency Things are done for rather than with older people There is a focus on completing tasks rather than enabling older people Physical environments do not always support older people	

#### 3.2.1 Challenges

##### 3.2.1.1 Risk Aversion

Participants noted how a risk‐averse culture can prevent staff members from using their initiative to trial new practices, stifling the innovation needed to promote brilliant care for older people. They indicated that older people were seldom offered choice, particularly if those choices posed potential risks. As one participant lamented, older people were not always encouraged to engage in simple pleasures, like taking a stroll—instead, staff members sometimes offered the use of a wheelchair to reduce the risk of a fall. Yet, this risk‐averse approach diminished older people's personal agency:

for some reason in the geriatric and dementia world, we've become complacent with the ‘no can do’ and be very risk averse and looking at why we can't do something first, as opposed to looking at all the different possibilities and really daring to think differently (*professional*).

##### 3.2.1.2 Limited Personal Agency

Related to risk aversion, several participants commented that, in care settings, tasks were often done for older people, rather than by or with older people, limiting their personal agency. Older people were denied simple acts, like dressing or picking up a dropped item. In the context of a risk‐averse culture, this was partly due to a task‐focused ethos, whereby care for older people was merely a job comprised of tasks that required completion. Staff members were required to shower, dress, and feed clients within an established timeframe—failure to do so might result in dire consequences for the staff member and the service. This often made it difficult to create environments that enabled older people to exercise personal agency and promote their independence. As such, when older people came to reside in a facility, much of the incidental physical activity in their daily routine disappeared. This in turn diminished older people's personal agency and physical capabilities, as well as staff members' ability to offer personalised care:

when the organisation promotes… person‐centred care… you actually look at what's happening. It's that lack of understanding in that culture in the workforce, that they're not really doing that—they're just very task‐focused (*professional*).

Similarly, lived experience experts noted the importance of personal agency, whereby older people were supported to do what they preferred, when they preferred. For instance, one participant recounted the innovative care that her husband—who lived with dementia—received at a residential facility. Previously employed as a ‘top administrator in the public service’, her husband once managed ‘an office with a lot of people’. After moving into the facility, he enacted managerial practices with props sourced from the facility's office, including the sign‐in book. Because the sign‐in book could not be removed from the office, a staff member found novel ways to encourage personal agency, which the participant appreciated:

[my husband] was taking the sign‐in book from the front office, taking a cushion from one of the chairs which had a zip cover; he would put the sign in book in this cushion with the zip cover, and walk around the facility with this cushion under his arm… he was checking on his staff and then… scribble on the… sign‐in book because they were his notes… the lifestyle officer spoke… said, ‘We've got to stop him taking this book… why don't you bring a briefcase in from home. I will buy a journal; I'll buy some pens and every day, I'll sit down with your husband and we'll have a meeting and he can write his notes in this journal’… she would sit with him, have a meeting; he would scribble his notes; he would put the journal in his briefcase, and he would carry that round… it was a case of thinking outside the square. There's no way you'll get him to play bingo or any of those activities. He wanted to walk around the facility and check on his staff. So that's the… innovation… [There] really wasn't any effort as far as the time of the diversional officer, because she would have been spending time with him anyway; it was just a case of, what can I do during that time? (*lived experience expert*).

##### 3.2.1.3 Impersonal Care

According to the participants, within a risk‐averse culture, where task completion reigned, staff members typically had little opportunity to personalise the care they offered to older people and their family members. When care for older people was recognised as merely a job, participants indicated that staff members were often unable to develop meaningful relationships with older people and their family members to better understand and attend to their needs and preferences. Instead, staff members awarded primacy to the tasks they were required to complete, irrespective of whether older people and their family members wanted this. This was partly due to the different ways that professional boundaries were understood and enacted within the sector offering care to older people. For instance, reflecting the risk‐averse culture, some participants spoke candidly about staff members who enacted rigid professional boundaries that disconnected staff members and older people, ensuring staff members ‘don't get close to anybody’ (*lived experience expert*)—this in turn, had dire consequences:

one of the big challenges… is the absolute resistance on the part of the agencies to allow or to give their care workers any opportunity to form relationships with the people that they're caring for… I get care workers come in and out all the time and some of them are fantastic and some of them are… terrible… there's… very tight boundaries about… what they're allowed to do with you or for you and what they're allowed to talk about… they're always told to set up set boundaries, to behave professionally… because then you'll just be really upset… when they die… professional boundaries means… you don't get too close to them [or]… the family. You don't go to funerals… [which is] completely unreasonable. It's like you're a prison officer, not a… care worker… It's… a task orientation (*lived experience expert*).

Yet, others noted that, in some organisations, a professional boundary was understood and enacted differently. Specifically, it was not synonymous with impersonal care—but rather, it served to demarcate an appropriate relationship between staff members and older people, ensuring safety, trust, and effective communication. These participants indicated that a professional boundary upheld ethical standards, clarified roles and responsibilities, and helped to prevent burnout by maintaining a healthy work‐life balance—this was particularly important given limited workforce capacity in the sector offering care to older people. As such, a professional boundary did not prevent personal connections; but rather, it bolstered it by sustaining staff members' well‐being. While they acknowledged the difficulty of establishing and maintaining a professional boundary, it was deemed essential to enable staff members to be present with older people while at work, and be present with family members while at home:

I have been a care worker for many years and now in management… there have to be some boundaries… to protect both staff and the clients… people who work in this industry are naturally carers—they're givers; they want to give… I'm not saying they can't develop relationships, but… they… need to have some boundaries on those relationships, because they will end up giving… it's a really tricky thing… to get that right level of boundaries and relationship… and if they don't get it right, they can end up giving up their own time and doing things for clients after hours they're not paid for, which then eventually affects their personal and family lives as well (*professional*).

While participants did not agree on how professional boundaries were understood and enacted within the sector, they did agree that impersonal care had implications for brilliant care for older people. This was largely for two reasons. First, impersonal care hindered a deep understanding of older people. Beyond attending to an older person's clinical and personal care needs, staff members typically had limited opportunity to get to: know an older person; understand their emotional and social needs and those of their family members; and tailor care accordingly. Second (and relatedly), impersonal care perpetuated the mindset that care for older people cannot be more than a job. This in turn can exacerbate disengagement among staff members, whereby they become demoralised.

##### 3.2.1.4 Low Morale and Energy

Participants agreed that the workforce that cared for older people was often beleaguered by low morale and energy. Staff members were overburdened and worn down, depleting their morale, energy, and capacity to deliver the type of care that older people needed and wanted. As such, low morale and energy contributed to a vicious cycle, whereby negativity begot negativity. According to the participants, this was partly due to the short supply of resources, including time, and the continued pressure to do more with less:

they become worn down and tired… they've been doing extra shifts… people forget when they get tired, why they came to aged care (*professional*).

#### 3.2.2 Principles

##### 3.2.2.1 Connection

To address some of the aforesaid challenges, participants reinforced ways to promote connection. This largely involved person‐centred care (Nilsen et al. 2022) and the person‐to‐person experience of care for older people, their family members, and staff members. The participants described ways to humanise the experience of care to demonstrate that everyone matters. This had the potential to create opportunities to develop a deep understanding of the older person and recognise that care for older people is not solely a job, but a relationship:

working in a clinical healthcare setting, people can be reduced to being patients, which is dangerous, or in the residential sector, to being residents, as opposed to them being themselves… if we start with… [a] ground up approach… that leads into that ongoing, sustained dialogue… if we see everyone as a person… then they will emerge (*clinician*).

To foster connections, the participants recognised the need to situate the older person at the centre of decisions. They spoke of ways to ensure the older person had the dignity of choice to exceed their expectations of care. This involved getting to know the older person, understanding what is meaningful to them and what offers them a sense of purpose, and attending to their needs and preferences:

we need to… have that dignity of choice… letting people out and about in the community. Yeah, they might get lost, but they also might not. So… really giving people choice of what… they want (*professional*).

The participants identified practical ways to promote connection. For instance, meaningful person‐centred assessments would allow staff members to develop a detailed understanding of an older person's life story and lifestyle. Rather than focus solely on clinical needs, deficits, and challenging behaviours, the participants spoke of the need to focus on what matters most to an older person.

##### 3.2.2.2 Innovation

Complementing their principles to promote connection, the participants described those to promote innovation—that is, different ways of working that positively deviated from norms within the sector. For instance, to develop a deep understanding of the older person, they suggested the need to develop and sustain dialogue with older people and within their circle of care—this would involve regular conversations between older people, their family members, and the staff members who support them. Given that different people were likely to be involved in the care of an older person and their family members, the participants also recognised the need for practical methods to optimise communication—these might include online platforms or handwritten notes to ensure a shared understanding of what the older person needed and wanted. While some of these innovations might seem obvious, the participants attested they did not always occur within services that offered care to older people, but should, given their feasibility:

setting up a book, before a resident comes into an aged care facility. Some people call it the life book, which covers not only medical history, but something that their relatives can complete before the person comes in providing photos, stories, interests, all that sort of thing (*lived experience expert*)

To recognise that caring for older people is ‘more than a job’, participants identified feasible ways to change the status quo in the sector. Similarly, the participants noted viable ways to ensure staff members within the sector could reprioritise what they did and how they did it. For example, encouraging older people to walk short distances or participate in their own personal care would empower them to reclaim independence, maintain physical and cognitive abilities, and engage and interact with different staff members. This reflected participants' suggestion to make care for older people everyone's business. This would involve encouraging all staff members—including ancillary staff members—to care:

anyone that walks through the door, whether it be a secretary, a person doing laundry, whether it be a cleaner that's working in aged care, no matter who it is, everyone should be able to deliver care in an aged care environment (*professional*)

### 3.3 Model of Brilliant Care for Older People

**FIGURE 2:**
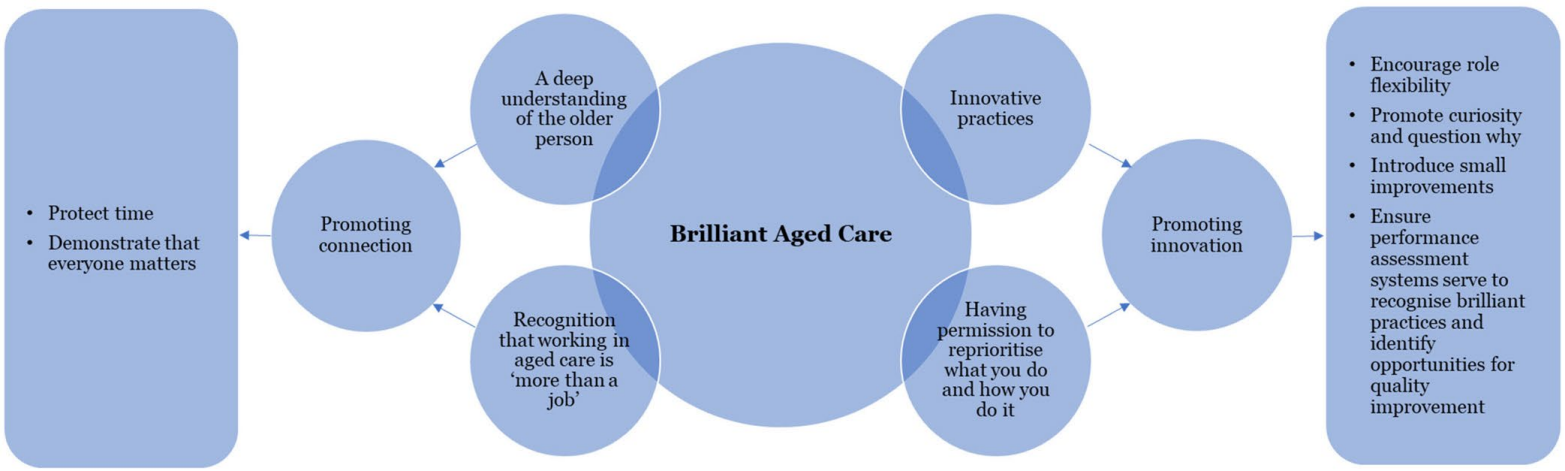
Model of brilliant care for older people. [Colour figure can be viewed at wileyonlinelibrary.com]

Having identified feasible principles to address the challenges and enact brilliant care for older people, the participants were invited to prioritise these through discussion and agreement during the workshops (see Appendix 1) to inform a model of brilliant care for older people (see Figure 2). This required the participants to identify three principles the aged sector should focus on and determine how these should be operationalised. While some participants spoke to their preferred principles, others noted these in written form via the online platform.

To promote connection, the participants collectively prioritised: protecting time; and demonstrating that everyone matters. The former referred to the intentional management and preservation of a staff member's time to ensure it was directed to the constituents of brilliant care for older people. Participants described a need to value time as a finite resource and exercise measures to guard against its wasteful or unproductive use—that is, practices that detracted from brilliant care for older people. Demonstrating that everyone matters was to recognise and respect the inherent value and worth of every individual, regardless of whether they received or delivered services, and regardless of their position or standing. According to the participants, it involved treating others with dignity, empathy, and fairness, and actively working to ensure that everyone's needs, perspectives, and contributions were acknowledged and considered.

To promote innovation, the participants prioritised: encouraging role flexibility; promoting curiosity and questioning why; introducing small improvements; and ensuring performance assessment systems serve to recognise brilliant practices and identify opportunities for quality improvement. Encouraging role flexibility referred to promoting a mindset and, relatedly, an organisational culture that values versatility, adaptability, and openness to performing different roles or tasks, as required. It involved rethinking what it means to care for older people and who is permitted to deliver care. According to the participants, this required a breakaway from rigid job descriptions or traditional boundaries and embracing a more fluid approach to work responsibilities. According to the participants, promoting curiosity and questioning why, involved fostering a culture of exploration and critical thinking. In a service that epitomised this principle, staff members were encouraged to challenge assumptions, seek deeper understanding, and pursue innovative solutions. Embracing curiosity fuelled growth, empowering individuals to engage with challenges with creativity and insight. Additionally, participants noted the importance of introducing small improvements. This principle served to normalise innovation—making incremental changes to processes and practices reinforces the importance of continuous improvement, potentially alleviating the concern or anxiety often associated with large‐scale change. Furthermore, the participants suggested that small improvements would likely beget small improvements, whereby staff members would readily engage with innovation. Finally, the participants prioritised efforts to ensure performance assessment systems recognised brilliant practices and identified opportunities for quality improvement. For instance, award schemes might be used to raise the profile of brilliant care for older people, as demonstrated by an individual, a team, or a service. Similarly, performance assessment systems might be used to identify opportunities to bolster brilliant care for older people, rather than castigate staff members.

## 4 Discussion

As the demand for and international importance of care for older people grows (United Nations 2001; World Health Organization 2022), so too does the negative discourse about care for older people (Blackberry and Morris 2023; Manchha et al. 2023). This in turn can have implications for patients, carers, clinicians, health services, and policymakers (Barney et al. 2009; Butterill et al. 1992). Building on research that critically reconceptualises older people and care for older people (Kontos et al. 2017), as well as that which established the constituents of brilliant care for older people (Dadich et al. 2023), this article serves to redress the imbalanced focus on all that is wrong with care for older people by presenting a model of brilliant care for older people. Co‐designed with stakeholders who had a stake in this study—including lived experience experts, managers, professionals, clinicians, and an academic—the model clarifies how brilliant care for older people can be feasibly realised.

Following three co‐design workshops, the co‐designed model of brilliant care for older people is comprised of principles to promote connection and those to promote innovation. The former includes protecting staff members' time for brilliant care for older people and demonstrating that everyone matters, irrespective of their role or position in the sector offering care to older people. The latter includes encouraging: role flexibility, curiosity, small improvements, and the recognition of brilliant practices. While the strategies to operationalise these principles within a particular service would require a consideration of context—including (but not limited to) the organisational culture—collectively, they have the potential to address some of the identified challenges that stymie brilliant care for older people. For instance, impersonal care and low morale and energy might be addressed by promoting connection—specifically, this might involve appreciating individuals, as well as awarding and recognising brilliant care (see Appendix 1). Similarly, risk aversion and limited personal agency might be addressed by promoting innovation—specifically, this might involve encouraging physical activity in its varied and routine forms.

Despite the value of the model of brilliant care for older people, five methodological limitations warrant mention. First, the small number of participants limits the transferability of the findings. Second, the sole involvement of participants who were conversant in English and could contribute to this study via web‐conference might have introduced selection bias—this is because the experiences and perspectives of people whose primary language is not English and who do not regularly use web‐conferencing might be under‐represented. Third, the cross‐sectional study design and reliance on self‐reported data make the findings specific to the historical context in which the study was undertaken. In particular, the COVID‐19 pandemic and the Royal Commission into Aged Care Quality and Safety (2021) revealed ‘systemic weaknesses’ (Cousins 2020, p. 1322) in Australia's care for older people, intensifying criticisms about the sector during this period—however, given the expressed focus of this study, it is unclear how these key events affected the study participants. Fourth, given the varied understandings of co‐design processes (Masterson et al. 2022), the use of an alternative co‐design process and the involvement of different individuals might have culminated in a different model of brilliant care for older people. Fifth, despite the similarities between care for older people in Australia and that in other nations, including the importance of homely facilities (Jain et al. 2019), the model might not be readily transferable further afield, given the recruitment method, which restricted participation to individuals in Australia.

Notwithstanding the aforesaid limitations, this article has implications for scholars. While there is the customary call for more research, what is specifically required is clarity in two areas. The first pertains to the utility and effectiveness of the co‐designed model of brilliant care for older people and its use among people who work in the sector (Bedenik et al. 2024). This might involve a process and an impact evaluation of the model across different care settings in Australia, such as residential, short‐term and home care—while a process evaluation might assess the implementation of the model, an impact evaluation might establish the associated effects at the micro level (for older people, their family members, and staff members), the meso level (for organisations and/or the services, therein), and the macro level (for communities). The second pertains to whether and how the model can be used in different nations.

## 5 Conclusion

This article presents a co‐designed model of brilliant care for older people, grounded in principles that foster connection and innovation. Central to this model is the recognition of older people's perspectives, which offer invaluable insights into what constitutes meaningful and effective care. By summarising the priorities of key stakeholders—particularly lived experience experts—the model not only validates their experiences but also underscores the relevance of these insights in shaping future care approaches. These principles can be implemented through practices that do not necessarily require large‐scale systemic change. While further engagement with stakeholders—particularly those who manage and deliver care—is essential to determine the resources needed for implementation, the model's potential is significant. This is especially true considering the increasing global demand for care for older people and the need to counter prevailing negative narratives surrounding ageing and care provision.

## 6 Relevance to Clinical Practice

Although the co‐designed model of brilliant care for older people presented in this article is yet to be tested, it has implications for clinical practice. For those who manage and deliver care for older people, the model encompasses simple, accessible, and cost‐effective principles to positively deviate from norms within the sector and deliver brilliant care for older people. Consider, for instance, the potentially inexpensive practice of treating others with dignity, empathy, and fairness or promoting curiosity and questioning why. Furthermore, given that the model was co‐designed with stakeholders, including lived experience experts, managers, professionals, and clinicians, its principles are imbued with their experiential insights, which served to bring particular priorities to the fore (Lowndes 2016).

## Author Contributions

A.D. conceived and designed the project, conducted interviews, as well as developed the introduction, methods and discussion sections. R.K. analysed the data and constructed the themes. A.D. and B.H.‐R. analysed data, informed theme construction and contributed to the development of the findings. All authors critically revised the manuscript and approved the final version to be published.

## Conflicts of Interest

The authors declare no conflicts of interest.

## Supporting information


File S1

## Data Availability

The data that support the findings of this study are not available due to privacy or ethical restrictions.

## Appendix 1 Priorities to Promote Brilliant Care for Older People

**Table:**

Identified Principles	Identified challenges			
	Risk aversion	Limited personal agency	Low morale and energy	Impersonal care
Promoting connection				
Let go of established practices				✓
Protect time			✓	
Appreciate individuals			✓	✓
Award and recognise brilliant care			✓	✓
Develop and sustain dialogue			✓	
Demonstrate that everyone matters			✓	✓
Shift norms about older people being at the centre of decision‐making				✓
Be older‐person‐centred				✓
Encourage human contact				✓
Introduce meaningful assessments so staff understand older people earlier				✓
Offer dignity of choice	✓	✓		
Reduce the precarity of secure employment			✓	✓
Recognise the experience of grief/loss				✓
Create affective encounters during transitions				✓
Remember that first impressions count				✓
Promoting innovation				
Encourage initiative				
Think differently	✓			
Ask questions beyond standard (clinical) assessment	✓			✓
Engage with (rather than avoid) risk	✓			
Encourage role flexibility	✓		✓	
Share ideas		✓		
Promote curiosity and question why		✓	✓	
Ensure the physical environment suits the needs and preferences of older people and family members		✓		✓
Introduce small improvements		✓		✓
Share different ways of working and make this the norm			✓	
Normalise initiative				
Encourage skill development				✓
Do not take no for an answer				
Share ideas about engaging with older people, service offerings and models of care				✓
Hire for culture			✓	
Ensure performance assessment systems recognise brilliant practices and identify opportunities for quality improvement			✓	✓
Encourage physical activity in its varied and routine forms	✓	✓		
