# Supplementary material for: Co‐Designing a Model of Brilliant Care for Older People
Source: J Clin Nurs. 2025 Aug 1;35(5):2468–82. doi: 10.1111/jocn.70049 (PMC13068167; doi:10.1111/jocn.70049)
Supplement: Supplementary file 1 — File S1 [file JOCN-35-2468-s001.docx]

# Research Reporting Checklist

Supplementary File 1: Consolidated Criteria for Reporting Qualitative Studies (COREQ): 32-item Checklist (Tong et al., 2007)

| **Nº** | **Item** | **Guide questions / description** | **Response** |
| --- | --- | --- | --- |
| **Domain 1: Research team and reflexivity** | | | |
| *Personal characteristics* | | | |
|  | Interviewer/facilitator | Which author/s conducted the interview or focus group? | Ann Dadich and Ben Harris-Roxas. |
|  | Credentials | What were the researcher’s credentials (e.g., PhD, MD)? | Ann Dadich holds a PhD and Ben Harris-Roxas holds a PhD. |
|  | Occupation | What was their occupation at the time of the study? | Both researchers are academics. |
|  | Gender | Was the researcher male or female? | Female and male. |
|  | Experience and training | What experience or training did the researcher have? | Both researchers had completed previous studies that involved: the collection, management, and analysis of qualitative data; as well as co-design processes. |
| *Relationship with participants* | | | |
|  | Relationship established | Was a relationship established prior to study commencement? | The participants were contacted via email to provide detailed study information and invite their involvement – furthermore, before each workshop begun, time was spent developing a rapport with the participants. |
|  | Participant knowledge of the interviewer | What did the participants know about the researcher (e.g., personal goals, reasons for doing the research)? | The participants were provided with detailed study information in written form – the researchers also verbally explained the focus of the study, its rationale, and the researchers’ roles in the study. |
|  | Interviewer characteristics | What characteristics were reported about the interviewer / facilitator (e.g., bias, assumptions, reasons and interests in the research topic)? | Ann Dadich pursues a research program on brilliant healthcare and Ben Harris-Roxas is a health services researcher – because these experiences might have influenced the workshop discussions and the analysis of the qualitative data collected during the co-design workshops, an additional researcher was involved in the analysis – namely, Rachael Kearns. |
| **Domain 2: Study design** | | | |
| *Theoretical framework* | | | |
|  | Methodological orientation and theory | What methodological orientation was stated to underpin the study (e.g., grounded theory, discourse analysis, ethnography, phenomenology, content analysis)? | A qualitative descriptive method, as per the approach espoused by Colorafi and Evans (2016). |
| *Participant selection* | | | |
|  | Sampling | How were participants selected (e.g., purposive, convenience, consecutive, snowball)? | Purposive sampling. |
|  | Method of approach | How were participants approached (e.g., face-to-face, telephone, mail, email)? | Via email. |
|  | Sample size | How many participants were in the study? | Thirteen participants contributed to the co-design workshops. |
|  | Non-participation | How many people refused to participate or dropped out? Reasons? | There were no participants who refused or revoked participation. |
| *Setting* | | | |
|  | Setting of data collection | Where was the data collected (e.g., home, clinic, workplace)? | The co-design workshops were facilitated via web-conference. |
|  | Presence of non-participants | Was anyone else present besides the participants and researchers? | No. |
|  | Description of sample | What are the important characteristics of the sample (e.g., demographic data, date)? | Of the 13 participants, most were female (*n*=11). The participants included: lived experience experts (*n*=5), managers (*n*=3), professionals (*n*=2), clinicians (*n*=2), and an academic (*n*=1). The three co-design workshops were facilitated a month apart in August, September, and October 2022. |
| *Data collection* | | | |
|  | Interview guide | Were questions, prompts, guides provided by the authors? Was it pilot tested? | Each participant was provided with a participant information sheet, which indicated the focus of, and justification for the co-design workshops. The workshop schedules were reviewed by the research team and the relevant human research ethics committee. |
|  | Repeat interviews | Were repeat interviews carried out? If yes, how many? | No. |
|  | Audio/visual recording | Did the research use audio or visual recording to collect the data? | Audio and visual data were digitally recorded. |
|  | Field notes | Were field notes made during and/or after the interview or focus group? | The researchers documented written notes during the workshop discussions. |
|  | Duration | What was the duration of the interviews or focus group? | Ninety minutes per workshop. |
|  | Data saturation | Was data saturation discussed? | Yes. |
|  | Transcripts returned | Were transcripts returned to participants for comment and/or correction? | The researchers documented written notes via an online shared whiteboard platform (Miro), which participants could access and edit. Although not all participants were involved in all three workshops, all participants received discussion summaries and were invited to confer with the facilitators between the workshops to ensure they were not disadvantaged by their absence. |
| **Domain 3: analysis and findings** | | | |
| *Data analysis* | | | |
|  | Number of data coders | How many data coders coded the data? | Two. |
|  | Description of the coding tree | Did authors provide a description of the coding tree? | No. |
|  | Derivation of themes | Were themes identified in advance or derived from the data? | Themes were derived from the data, as per the approach espoused by Colorafi and Evans (2016). |
|  | Software | What software, if applicable, was used to manage the data? | NVivo 12 Pro. |
|  | Participant checking | Did participants provide feedback on the findings? | The participants were invited to contribute to the development of the findings via three different strategies. First, during the three co-design workshops, the participants discussed and critiqued the findings constructed from the data. Second, the researchers documented written notes via an online shared whiteboard platform (Miro), which participants could access and edit, both during and between the three workshops. Third, all participants were invited to co-author the article to articulate, substantiate, and present the findings. |
| *Reporting* | | | |
|  | Quotations presented | Were participant quotations presented to illustrate the themes / findings? Was each quotation identified (e.g., participant number)? | Yes – however, participants were identified with an identification number, as per the protocol approved by the relevant human research ethics committee. |
|  | Data and findings consistent | Was there consistency between the data presented and the findings? | Yes. |
|  | Clarity of major themes | Were major themes clearly presented in the findings? | Yes. |
|  | Clarity of minor themes | Is there a description of diverse cases or discussion of minor themes? | Yes – this is presented within the themes, as appropriate (e.g., ‘Impersonal Care’). |

# References

Colorafi, K. J., & Evans, B. (2016). Qualitative descriptive methods in health science research. *HERD*, *9*(4), 16-25.

Tong, A., Sainsbury, P., & Craig, J. (2007). Consolidated criteria for reporting qualitative research (COREQ): A 32-item checklist for interviews and focus groups. *International Journal for Quality in Health Care*, *19*(6), 349-357.
